# Household outbreak of sporotrichosis: towards the One Health approach

**DOI:** 10.1590/0037-8682-0021-2022

**Published:** 2022-06-06

**Authors:** Elisa Helena Paz Andrade, Camila Valgas Bastos, Afonso Vieira da Silva, Simone Magela Moreira, Taiza Gonçalves de Araújo Costa, Lauranne Alves Salvato, Salene Angelini Colombo, Camila Stefanie Fonseca de Oliveira, Danielle Ferreira de Magalhães Soares, Kelly Moura Keller, Maria Isabel de Azevedo

**Affiliations:** 1 Universidade Federal de Minas Gerais, Departamento de Tecnologia e Inspeção de Produtos de Origem Animal, Belo Horizonte, MG, Brasil.; 2 Universidade Federal de Minas Gerais, Departamento de Medicina Veterinária Preventiva, Belo Horizonte, MG, Brasil.; 3 Centro Animal Céu Azul, Belo Horizonte, MG, Brasil.; 4 Instituto Federal de Minas Gerais, Departamento de Ciências Agrárias, Bambuí, MG, Brasil.; 5 Prefeitura Municipal de Contagem, Contagem, MG, Brasil.

**Keywords:** Contact tracing, Cat, Epidemiology

## Abstract

Although sporotrichosis requires a broad approach for control, few reports have described the relationship between the index case and secondary contacts. In the present work, we report an outbreak involving a woman, a dog, and two cats from the same household environment, including the clinical and epidemiological aspects and outcomes, and discuss the importance of a One Health approach to face this neglected disease. The joint efforts of professionals such as veterinarians and physicians are essential for early diagnosis and surveillance, which contributes to the rapid identification and control of zoonotic sporotrichosis outbreaks.

## INTRODUCTION

Sporotrichosis is a neglected subcutaneous mycosis caused by pathogens of the genus *Sporothrix,* which infect humans and animals. The infection has a global distribution, with several areas in the world, mainly Brazil^1^.

An increase in the number of human cases is usually preceded by an increase in the number of cats, which represents a serious public health problem^1^. Furthermore, in cats, skin lesions contain a high fungal load, resulting in high zoonotic transmission potential, which demonstrates the importance of this animal species in the epidemiological chain of sporotrichosis^2^. 

The One Health approach endorsed by the World Health Organization, World Organization for Animal Health, and various bodies at international and national levels constitute a transdisciplinary model of human, animal, and environmental health^3^. Therefore, combining the spread of cat-transmitted sporotrichosis with a One Health approach will require multisectoral efforts, including veterinarians, physicians, epidemiologists, microbiologists, environmental scientists, and many other partners^4^.

Exposure to infected individuals is the most important risk factor for communicable diseases. Therefore, studies on family members and close contacts of individuals infected with sporotrichosis are important sources of information. Furthermore, in household studies, only individuals who have been exposed are included, allowing for a better understanding of individual-level risk factors and quantification of transmission probabilities^5^. Thus, the present study aimed to report a series of four cases of sporotrichosis in the same residence and address the aspects of One Health to deal with the disease.

## CASES REPORT

A healthy 37-year-old woman from Minas Gerais, Brazil, reported being scratched on her right hand by a cat in September 2019. It was male, without a defined breed, surgically neutered, and had returned home with numerous body wounds, mainly on the head and paws, after a period of wandering the streets. Therefore, the cat was taken for veterinary care.

Cytological examination of the skin lesions was performed under a microscope, which revealed findings compatible with an intense pyogranulomatous inflammatory process and numerous structures with morphology compatible with *Sporothrix* spp. inside intralesional macrophages, resulting in the diagnosis of sporotrichosis. The animal was in poor general condition and was euthanized. The corpse was then incinerated. The veterinarian advised the woman to seek medical attention if her lesion in the hand did not heal as expected, due to the zoonotic character of sporotrichosis.

**FIGURE 1:**
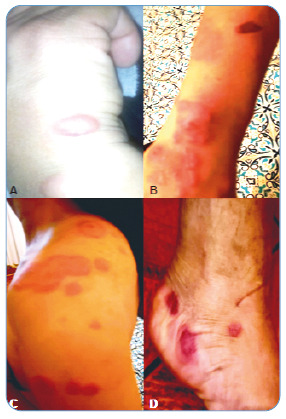
Lymphocutaneous form of sporotrichosis manifested by a previously healthy 37-year-old woman after a cat scratch. The skin lesions started at the right hand (site of trauma) as well-defined red and swollen areas, with the aspect of a pimple **(A)**, and then spread to other parts of the body, including the right forearm **(B)**, left shoulder **(C)**, and left foot **(D)**.

Twenty-one days after the scratch, the woman identified an injury at the trauma site and visited a physician in October 2019, who made a clinical and epidemiological diagnosis of sporotrichosis based on the history presented. As an early sign, the woman noticed redness, swelling, and a fixed localized lesion, followed by other pimple-like lesions. Days later, other lesions on the forearm, arm, shoulder, and lateral and contralateral scapula were observed, which is characteristic of the lymphocutaneous form. Subsequently, the injuries reached the legs and feet (Figure 1). Itching at the lesion sites has also been reported. Oral itraconazole 200 mg/day for 1 month and 100 mg/day for an additional month and a half were prescribed for treatment. The wounds improved dramatically with clinical cure after 2 and a half months of treatment.

**FIGURE 2:**
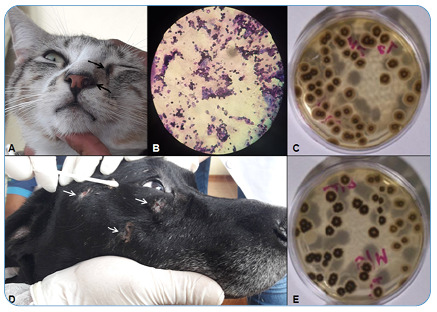
Skin lesions, sample collection, and results obtained from the second cat and dog. **(A)** The skin lesions on the muzzle and lower medial border of the left eye are indicated by arrows. **(B)** Cytology of the cat’s skin lesion imprint stained with panoptic kit and viewed using a microscope showing the presence of yeast forms. **(D)** The dog’s skin lesions were characterized as ulcerated and crusted areas of alopecia on the head (indicated by arrows), of which one was chosen for sample collection with a sterile swab for fungal culture. **(C and E)** Growth of *Sporothrix* spp. in the fungal culture from skin lesions of the cat and dog, respectively.

Approximately 2 months after the diagnosis of cat sporotrichosis (index case), a dog and another cat that shared the same domestic environment were taken for veterinary care. The cat was male, mixed-breed, not surgically neutered, and had initial skin lesions on the muzzle and left eye's lower medial border (Figure 2A). Impressions stained with a panoptic kit were prepared from the cat lesions. Oral amoxicillin with potassium clavulanate (15 mg/kg) was prescribed for 12-12 hours until the result. Cytology revealed the presence of yeast in the lesions (Figure 2B). The male, mixed-breed, non-surgically neutered dog presented with ulcerated skin lesions on the head with crusted areas and alopecia (Figure 2D). The dog was serologically negative for canine visceral leishmaniasis. Skin lesion samples were collected with sterile swabs from the two animals for diagnosis at the Mycology and Mycotoxins Laboratory of the Federal University of Minas Gerais. There was a growth of *Sporothrix* spp. in fungal culture for both (Figures 2C and 2E), confirming sporotrichosis. *Sporothrix* species isolated from the dog and second cat were identified as *Sporothrix brasiliensis* by polymerase chain reaction (PCR) based on calmodulin gene sequences^6^.

Oral itraconazole 100 mg/day and 180 mg/day for 60 days were prescribed with liver protector for the cat and dog, respectively. Additional precautions were recommended, such as separating these animals from others in the household and not allowing access to the street.

After a month of treatment, in January 2020, the cat returned to the veterinary clinic and still had nasal edema. Oral potassium iodide (8 mg/day) associated with itraconazole (100 mg/day) was prescribed. In the same month, the dog was euthanized because of the worsening of its general condition and comorbidities. The cat returned to the veterinarian in March 2020 after 50 days of treatment with potassium iodide and itraconazole, and its clinical cure was confirmed.

**FIGURE 3:**
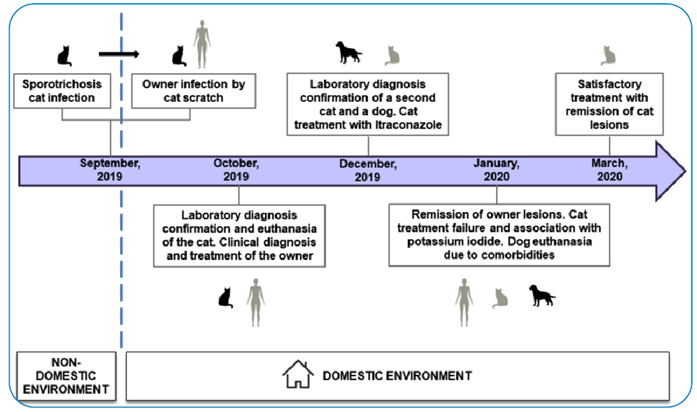
Timeline of the main events involving the transmission, diagnosis, treatment, and outcome of the owner and animals with sporotrichosis in the same household environment.

The main events related to the transmission, diagnosis, treatment, and outcome of sporotrichosis in human and animals are summarized in Figure 3.

## DISCUSSION

Household studies provide a unique opportunity to determine the transmission of infectious diseases^5^. In the present study, a cat that had access to the street (index case) was the primary source of *Sporothrix* spp. in the residential environment, transmitting this agent to its owner through scratching. Two other animals (a dog and another cat) were also infected, probably by direct contact with the first cat, as these animals had no access to the street. *S. brasiliensis* was detected in the dog’s and second cat’s skin lesions, probably being the species responsible for the outbreak.

Despite being neutered, access to the street represented an important element of risk, contributing to infection in the index case. In the same region, there is a three times greater probability of a positive diagnosis of sporotrichosis in cats with street access^7^. Another study showed that wandering freely on the streets is a significant risk factor for *Sporothrix* spp. infection in cats, even after desexing^8^.

In a restricted environment, such as in the same home, the risk of a dog being infected by contact with a sick cat is high; however, we believe that the dog has a limited epidemiological role because canine lesions have few fungal microorganisms^9^ and probably it is not involved in the transmission to the second cat in the present study. 

The owner's infection occurred through scratching of the first infected cat, which characterizes zoonotic sporotrichosis. The patient's clinical diagnosis was well defined since the patient had a detailed clinical history. The characteristics of the lesions were consistent with the lymphocutaneous form, the most common clinical presentation^10^, and lesions regressed with itraconazole treatment. The combination of itraconazole/potassium iodide showed better results in the second cat. The effectiveness of both drugs as monotherapy has been documented, but cases of therapeutic failure are common in cats^11^.

Early diagnosis and surveillance are essential to facilitate rapid identification and control outbreaks between cats and humans^11^. In addition, public strategies to educate the population about responsible feline ownership and aspects of zoonotic sporotrichosis transmission are essential to limit the spread of this emerging and re-emerging disease^1^ ^,^ ^12^. 

In a One Health context, veterinarians are needed to address the spread of *S. brasiliensis* among the feline population and investigate other potential host species. Their roles should include the treatment of infected animals, education of pet owners on the potential for zoonotic exposure, and promotion of appropriate husbandry and mitigation efforts necessary to limit the spread of *S. brasiliensis*. When educating clients and discussing the treatment of feline sporotrichosis, veterinarians need to highlight the difficulties of treatment, including the need for long-term adherence to an antifungal regimen, the risk of adverse reactions to these medications, and the need to keep infected cats indoors to prevent the spread of this fungus during treatment. Additionally, when owners decline treatment or treatment fails, veterinarians will need to discuss euthanasia and cremation, as releasing infected cats or burying the remains of their pet could introduce fungal spores into the environment and perpetuate the spread^4^. 

Physicians should be alert for cases of cat-transmitted sporotrichosis, request proper diagnostics, and ensure rapid initiation of antifungal therapy. Physicians and veterinarians should also collaborate on the investigation of suspected zoonotic outbreaks, development and enhancement of surveillance systems for human and feline sporotrichosis, and research into One Health technique for controlling cat-transmitted sporotrichosis^4^. In the reported cases, access to veterinary services was essential to consolidate the infected animals and the owner´s diagnosis.

Our study highlights the potential for intra-household sporotrichosis transmission. Considering the context in which cats have access to streets, applying strategies to mitigate the disease and prevent transmission within families is necessary. In addition, new models of interventions in public health based on a One Health approach should prioritize cases and high-risk contacts, whose information will provide a better understanding of the extent, nature, and determinants of transmission in restricted environments. 
